# Effectiveness of Nanolime as a Stone Consolidant: A 4-Year Study of Six Common UK Limestones

**DOI:** 10.3390/ma12172673

**Published:** 2019-08-22

**Authors:** Stelios Tzavellos, Giovanni L. Pesce, Yu Wu, Alison Henry, Simon Robson, Richard J. Ball

**Affiliations:** 1 BRE Centre for Innovative Construction Materials, Department of Architecture and Civil Engineering, University of Bath, Bath BA2 7AY, UK; 2 Department of Architecture and Built Environment, Northumbria University, Newcastle upon Tyne NE1 8ST, UK; 3 Building Conservation & Geospatial Survey, Technical Conservation Department, Historic England, The Engine House, Fire Fly Avenue, Swindon SN2 2EH, UK

**Keywords:** nanolime, limestone, drilling resistance, consolidation

## Abstract

Protecting stone buildings from weathering and decay is a major challenge in the conservation of built heritage. Most of the stone consolidants currently available are well suited to silicate stones, but are less compatible with limestone. In this paper we present for the first time the results over a 4-year period of various consolidation treatments carried out using nanolime on 6 of the most representative and significant stones used in historic buildings in the United Kingdom. Tests investigated the influence of stone type, environmental conditions and pre-treatments on the effectiveness of the consolidation treatment. A comprehensive and rigorous testing programme was carried out to evaluate the short (12 weeks) and longer-term (4 years) effects. Stone samples were characterised before and after treatment using light and electron microscopy, sorptivity tests and a novel methodology employing drilling resistance to interrogate the near surface effects. Results show that for some of the stones, such as Clunch and Bath Stone, the positive effect of the treatment with nanolime is noticeable after 4 years since application. However, results for other stones such as Portland and magnesian limestone showed that the initial beneficial effect of the treatment is reduced after 4 years. Nanolime treatment of Ham Stone produced an unnoticeable effect on the continuous natural reduction of the drilling resistance of the specimen over time. The results presented are of immense value to conservators as they provide essential guidance on the most appropriate repair approach. Impact to the conservation industry will be to avoid the use of nanolime on stones where there is no perceivable benefit, reducing the risk of adverse effects, including potential damage to buildings. In additional costs will be saved which might otherwise have been spent on ineffective treatments.

## 1. Introduction

For centuries, types of limestone such as Bath and Portland Stones have been used as a building material in the United Kingdom. However, very often such stones are subject to decay that can alter their aesthetic, physical and mechanical characteristics and, in some cases, also their stability and the stability of the structure they belong to. Various mechanisms contribute to the stone decay. It is known, for instance, that in urban environments, atmospheric pollutants such as sulphur dioxide (SO_2_) and nitrogen oxides (NO_x_), and the resulting acid solutions produced in the atmosphere (i.e., acid rain) play an important role in accelerating the decay of limestone used in historic buildings. The sulphuric acid (H_2_SO_4_) contained in the rain dissolves the main component of the limestone (i.e., calcium carbonate or CaCO_3_) leading to the precipitation of gypsum (CaSO_4_·2H_2_O) which is a critical problem in the conservation of stones exposed to the weather, because its crystals are highly soluble and have a volume up to 30% larger than the volume of the original matrix where they can exert pressure on the pore walls inducing micro-cracking [1,2]. If the stones are exposed to cold and wet environments, freeze-thaw cycles can subsequently contribute to the degradation of the stone’s matrix, leading to weakening and eventually spalling of the stone surface or even shattering of entire blocks.

Stone consolidation as a method to reduce stones decay has been used for decades involving a variety of materials and treatments [3]. Ideally, all consolidants are required to have specific properties and performance characteristics. Consolidants should significantly improve the measurable properties of the decayed stone (such as compressive strength, elasticity, and abrasion resistance), have similar thermal and moisture expansion characteristics to the treated stone, and allow for future re-treatment with the same or a different consolidant. At the same time, it is important that no undesirable side-effects are caused by the treatment, as these could accelerate deterioration. For instance, consolidation treatment should not adversely affect the pore structure of the stone, nor the moisture transfer within it. Treatments should not cause strong interfaces between treated and untreated areas, nor form harmful by-products, or significantly alter the appearance (colour, texture or surface reflectance). Finally consolidation treatments should not encourage or support microbiological growth, nor affect its medium and long-term maintenance [3]. It is arguable whether any consolidants meet all the performance requirements listed above. For example, silane-based consolidants may affect the appearance, water absorption and porosity of the stone, and use of limewater may lead to mobilisation of harmful soluble salts.

The most common consolidants currently used include both organic and inorganic compounds able to stabilise and reinforce the stone’s weakened surface. These are, for instance treatments with silane-based consolidants such as ethyl-silicates and alkoxysilanes, acrylic polymers, and lime-based materials such as limewater and milk of lime. Silane-based consolidants are silica-based polymers with low-viscosity. They were first introduced to the conservation industry in the 1960s and, since then, conservators started to observe some negative effects such as changes of the natural colour of the stone, reduction of the stone porosity and in the water absorption [4]. Nevertheless, considering the limited consolidation effects of the alternative materials available, silane-based materials are still largely used. Acrylic polymers have been used as stone consolidants since the early 1960s. These are good transparent adhesives, stable against oxygen and UV radiation. However, acrylic products have limited penetration, are less water repellent compared to silicone polymers, and the water repellence properties can quickly degrade if the treated stones are subject to wet/dry cycles [5].

Lime based consolidants are among those that have been used for the longest time. Milk of lime is a suspension of micrometre-sized lime particles in water, whereas limewater is a clear solution of lime (calcium hydroxide) in water. Despite being chemically suited for treating limestones, the consolidation effect of these two materials is either limited, in the case of limewater (because of the very limited amount of calcium ions delivered with the solution: 1.7 g/L at 20 °C), or even negative, in the case of the milk of lime (because of the particle size is often too big for effective penetration inside the stone pores and therefore remain on the surface of the stone affecting its appearance). To improve performances of lime-based consolidants, in the late 1990s Baglioni, Dei and Salvadori synthesised nanometre-sized calcium hydroxide (Ca(OH)_2_) crystals to be used in the conservation and restoration of frescos [6]. This material is now commonly referred to as nanolime and over recent years it has been used as a stone consolidant in various countries, including the UK [2,7,8,9,10].

Nanolime consists of nanometersized Portlandite crystals suspended in alcohol. Typical particle sizes range between 50–600 nm, with regular shapes and crystalline features [11,12]. The most common product sold in the UK uses ethanol as a medium in which the crystals are suspended, but similar suspensions can also be produced using iso-propanol or *n*-propanol. Typical concentration of Ca(OH)_2_ is 25 g/L but suspensions with higher (e.g., 50 g/L) and lower (e.g., 10 g/L) concentrations are available too.

Theoretically, this material has potential benefits compared to other lime-based consolidants: nanolime crystals are much smaller compared to the particles contained in the milk of lime; the use of alcohol prevents the possible dissolution of water-soluble salts that may be contained in the stones, and improves the particle stability. Another advantage is that the nanolime products can carry larger quantities of Ca(OH)_2_ per litre compared to other lime-based consolidants such as limewater. Compared to other consolidants such as the silane-based ones, nanolime shows a reduced penetration. However, in contrast to the silanes (which do not form strong bonds with calcareous substrate [4]) nanolime is chemically compatible with limestone and is supposed to form strong bonds with it. In fact, similar to the milk of lime and limewater, the consolidating effect of nanolime is due to carbonation reaction (Equation (1)) in which water plays an important role, as it allows the dissolution of carbon dioxide (CO_2_) and the subsequent formation of carbonic acid (H_2_CO_3_) that leads to the precipitation of CaCO_3_ [13,14].

(1) $$\mathrm{Ca}{\left(\mathrm{OH}\right)}_{2\left(\mathrm{aq}\right)}+{\mathrm{CO}}_{2\left(\mathrm{aq}\right)}\to {\mathrm{CaCO}}_{3\left(s\right)}+H_{2}O_{\left(l\right)}$$

For this reason, the reaction is strongly dependent on various factors such as the residual water content of the stone and the concentration of particles used [15]. Research carried out by Dei and Baglioni [16] demonstrated that a time period of approximately 28 days is sufficient for the full carbonation of nanolime when used as a consolidant. However, the exact time is influenced by various factors such as stone type, its porosity, and the relative humidity of the atmosphere during application and curing. Nanolime products containing lower concentrations of Ca(OH)_2_ were shown to reach a fully carbonated state more rapidly than more concentrated ones [15]. This was attributed to the fact that with lower concentrations of nanolime particles are more widely spaced than in more concentrated forms, and thus expose a larger surface area to the air after evaporation of the solvent [15]. Daniele [11] found greater carbonation efficiency under higher humidity conditions due to the availability of water inside and on the surface of the treated stone, that allows the formation of carbonic acid and the subsequent reaction with calcium ions in solution. Dei and Salvadori [17] stated that nanolime can attain a deeper penetration when applied to limestone with low-porosity compared to treatments with larger particles such as the milk of lime. Work by Lopez-Arce [18] investigating the effect of treatment on porosity using ultrasound velocity measurements showed a reduction in pore sizes rather than complete filling. Through scanning electron microscopy (SEM) imaging of specimens before and after treatment, Daniele [11] also reported the partial filling of pores.

Overall, the research carried out to date suggests that, despite the theoretical advantages of nanolime when compared to other consolidants, the general beneficial effect of nanolime is still unproven. In some stones its limited effect can be explained by the accumulation of the Ca(OH)_2_ crystals on the surface of the stone, which is assumed to be due to the partial back-migration of particles during alcohol evaporation. Recent research from Borsoi [19] demonstrated that the resulting dense nanolime layer was probably impairing the quality of consolidation and limiting the penetration of successive treatment applications. Ongoing research attempts to improve the effectiveness of nanolime by optimizing the application procedure and using a range of different solvents. However, with the exception of the research funded by Historic England and carried out at the University of Bath [20], there has been very limited laboratory testing on nanolime treatment of naturally weathered stone in the UK [2]. This research contributes to filling this gap by investigating the influence of the stone type on the effect of the consolidation treatment as well as the effects of environmental conditions and of pre-treatments after 12 weeks and 4 years since treatment.

## 2. Materials and Methods

### 2.1. Materials

#### 2.1.1. Stones

**Figure 1:**
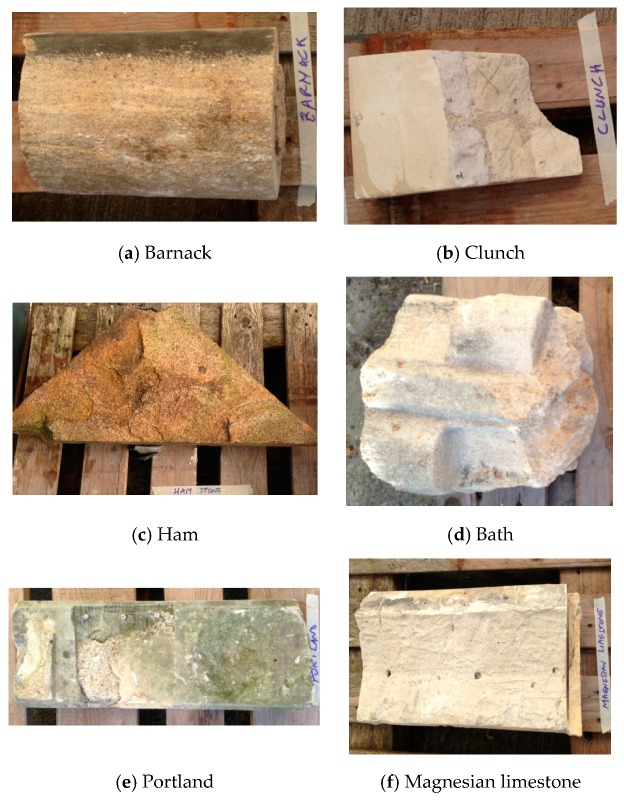
Images of the 6 different stone types used during the tests. In this image, the Barnack stone is represented only by the column used during the tests (**a**) whereas the Bath stone is represented only by one of the samples tested (**d**). The Magnesian limestone in the image (**f**) was split after the treatment. Sizes of stones can be estimated from the wooden slats of pallet in the background which are approximately 80 mm.

Nanolime treatments were applied to 6 different stone types representing some of the most commonly used lithotypes in the UK, namely clunch, Bath, Barnack, Portland, Ham and magnesian limestone (Figure 1a–f). Clunch (Figure 1b) is a limestone rich in clay and chalk formed during the Cretaceous period that was widely used in the past centuries in eastern England and Normandy. It is soft when quarried due to high water content, but following drying becomes progressively harder [21]. Bath Stone (formed during the same period; Figure 1d) is an ooidal sedimentary limestone consisting of granular fragments of calcium carbonate (called ooids) that was quarried in various places around the city of Bath (West of England) [22]. Barnack Stone (formed during the Jurassic period; Figure 1a) is an ooidal limestone from Lincolnshire (central England), sometimes called Barnack Rag, which has been valued as a building stone since the Roman times [21]. Of this stone, two specimens were used in this research, one was obtained from a carved (moulded) stone and the other from a column. Portland Stone (Figure 1e) is a well cemented ooidal limestone formed during the Tithonian stage of the Jurassic period and quarried on the Isle of Portland, in Dorset (South of England) [21]. This stone was widely used in the past all over the UK and is characterized by a good resistance to weathering whilst remaining soft enough to be worked by masons. Ham Stone (Figure 1c) is a medium to coarse-grained shelly Jurassic limestone from the Toarcian or upper Lias stage, obtained from Ham Hill quarry in Somerset (West of England) [21]. The Magnesian limestone (Figure 1f) used in this research dates from the Permian period and is sourced from a suite of rocks in the North-East of England. Unlike the other litothypes used in this study, the Magnesian limestone mainly consists of dolomite which is a calcium and magnesium carbonate [21].

All stones were sourced from various buildings and monuments in England and therefore their conditions at the beginning of the tests were typical of weathered stones. This included organic growth and, in some cases, the formation of a friable surface layer, or the formation of a surface crust produced by the dissolution and re-precipitation, within the pores, of new mineral phases [23].

For each stone, only the exposed surface (weathered) was used during the tests. Since each stone was different (i.e., ashlar, pieces of column), the tested surfaces had different characteristics (e.g., some surfaces were flat whereas others where curved), however, since all stones were sourced from various buildings, all surfaces were finely worked. Often the geometry of the stone dictated the location and direction of the tests such as the location and direction of the holes for the drilling resistance measurements. Testing locations were selected which exhibited visually similar weathering and stone surface texture. The dimensions, shape, colour and texture of the stones is shown in Figure 1.

#### 2.1.2. Nanolime

The nanolime used in these tests was a commercial product called CaLoSiL^®^ E25 produced by the German company IBZ—Salzchemie GmbH & Co. KG (Halsbrücke, German) [24] and supplied to the UK’s conservation market by Hirst Conservation Ltd. (Sleaford, UK) The product is a suspension of nano-portlandite crystals in Ethanol with concentration of 25 g per liter of alcohol. The product was used as received. Only newly supplied nanolime, well within the use-by-date, was used during the treatment of the stones. 

### 2.2. Methods

To reproduce the treatment to which stones are often subjected during on-site restoration works, before any consolidation treatment was applied, all stone samples were cleaned by a conservator using a common steam cleaner and a stiff brush. This helped removing surface contaminants, biological growth and loose material. After cleaning, excess of water was removed allowing the specimens to equilibrate with the conditions of an environmental chamber where the temperature varied between 18 and 20 °C and the relative humidity between 55% and 75%. Subsequently all specimens were characterised using a combination of complementary techniques to elucidate the stone structure, sorption characteristics and mechanical properties of the surface.

During treatment, all specimens were positioned so that the surface to be treated was vertical to simulate application on a wall. Using a syringe, nanolime was flooded slowly (trying to avoid too much run off) over the surface of each specimen until no more product was absorbed (absorption was assessed visually). The method used in the laboratory reproduced as closely as possible the application methods used on-site by professional conservators. The application was repeated at 24-h intervals over a period of 6 days. Details of the treatments are reported in Table 1. The application took place under laboratory conditions at a temperature of 20 °C and relative humidity between 50–70%.

After treatment, all specimens were stored for 12 weeks in an environmental chamber under the same conditions as described above. At the end of this period the mechanical properties and the sorption characteristics of all specimens were tested again. Subsequently, all stones were stored outdoors for 4 years in an open cabinet protected from rain. During outdoor storing, the temperature fluctuated between −1 to +20 °C and the relative humidity varied between 40% and 100%.

To investigate the effects of environmental conditions on the consolidation treatment, the Magnesian limestone was split into two pieces immediately after the treatment with nanolime. During the initial 12 weeks a part of this specimen was stored in the environmental chamber, whereas the other part was stored outdoors under similar conditions to those described above. After the initial 12 weeks both pieces were stored outside as with all other specimens.

To investigate the effect of pre-treatments on the consolidation effects of nanolime, three different specimens of Bath stones produced from the same block were used: Specimen #1 was pre-wetted with water in order to promote penetration of the nanolime; Specimen #2 was not pre-wetted, and specimen #3 was pre-wetted with ethanol (i.e., the same alcohol used as carrier in the nanolime).

### 2.3. Characterization Techniques

To investigate the characteristics of untreated stones, complementary microscopy techniques were applied to polished sections of representative fragments of all stones including the exposed surfaces and some areas representing the bulk. The polished sections were prepared by vacuum impregnating fragments of the various stones with low-viscosity resin followed by curing of the resin at a pressure of 0.34 MPa for 12 h. The section of each specimen was subsequently ground using progressively finer silicon carbide papers to a 1200 grit size before polishing with 6, 3 and finally 1-micron diamond paste. A final polish was achieved using a colloidal silica suspension. The sections were, then, examined using a Zeiss ICM 405 metallurgical microscope (Oberkochen, Germany), a JEOL 6480 LV Scanning Electron Microscope (SEM) (Tokyo, Japan). To provide a visual record of the stones’ morphology and pore size near the surface, 4 years after the treatment a JEOL FESEM6301F field emission SEM (Tokyo, Japan) was used. For SEM analysis, all sections were coated with chromium to minimise surface charging during analysis. All images were captured at a magnification of 10k times.

#### 2.3.1. Porosity Changes

**Figure 2:**
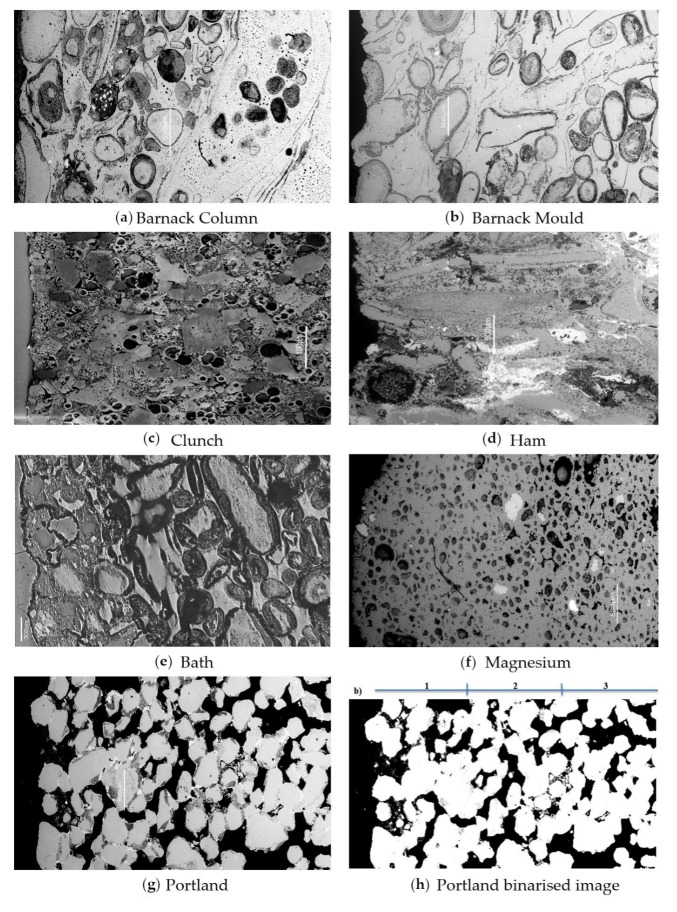
SEM cross-sections of the stone specimens. (**a**) Barnack Column, (**b**) Barnack Mould, (**c**) Clunch, (**d**) Ham, (**e**) Bath, (**f**) Magnesian, (**g**) Portland, (**h**) Portland image with adjusted threshold showing pores in black and stone in white (5200 µm × 2500 µm in ×50 magnification). Surface of stone shown on left hand side of image with 1, 2 and 3 indicating regions of increasing depth from the stone surface.

To investigate porosity changes within the sections, backscattered electron images of the specimens before treatment and after 12 weeks treatment were acquired and imaging analysis techniques were applied [25,26]. The software used for the analysis was ImageJ, version 1.46 r [27]. Following calibration, images were binarised by applying a shading correction median filter and adjusting the threshold. Each image was then divided into three regions where the porosity was calculated: region #1 included the surface of the specimen and the area immediately behind it; region #3 represented the deepest part of the sample inside the stone (bulk); and region #2 is the region between region #1 and region #3. The original images were consulted throughout this process to ensure that the binarised images correctly reflected the porosity of the stone. An example of a typical image before and after processing is shown in Figure 2g,h, respectively.

#### 2.3.2. Drilling Resistance Measurements

To investigate the effects of the consolidant at various depths from the treated surface, measurements of the drilling resistance (DR) were collected [2]. A SINT Technology Drilling Resistance Measurement System (DRMS) equipped with a 5mm diamond-coated flat-tipped drill bit was used to measure the DR of the stone before treatment and after 12 weeks and 4 years following treatment with nanolime. To take account of the natural variability of the stones, for each specimen 3 to 7 holes were drilled up to a depth of 40 mm and the results of the 3 most consistent measurements were averaged to provide a representative resistance profile. To acquire representative DR profiles, holes were drilled in different parts of the treated surface. A rotational speed of 600 rpm and a penetration rate of 10 mm/min were used in all tests [28]. Considering the low drilling resistance of all stones, no calibration was adopted to correct for the wear of the drill bit [28]. To determine whether natural weathering had an effect on the mechanical characteristics of the stones over the test period (which might in turn counteract any consolidating effect due to the nanolime treatment), the DR of untreated areas of the tested stones was also measured after 4 years.

#### 2.3.3. Water Absorption Test

Surface water absorption was evaluated using the Karsten Tube Penetration Test [2,29,30]. In each test the tube was firmly sealed to the stone surface using plasticine. Measurements were taken on the vertical face of the stone before treatment and then at 12 weeks and 4 years following treatment. The tube was filled with distilled water and the volume of water remaining in the tube during absorption was recorded at time 0 and then after 3, 6, 9, 12, 15, 18, 21, and 25 min. Results of the water absorption test are presented as volume of water absorbed in millilitres against the square root of time (mL/s^0.5^). Sorptivity was calculated from the gradient of the line obtained, as described in reference [29].

## 3. Results

### 3.1. SEM Analysis and Porosity Change

**Figure 3:**
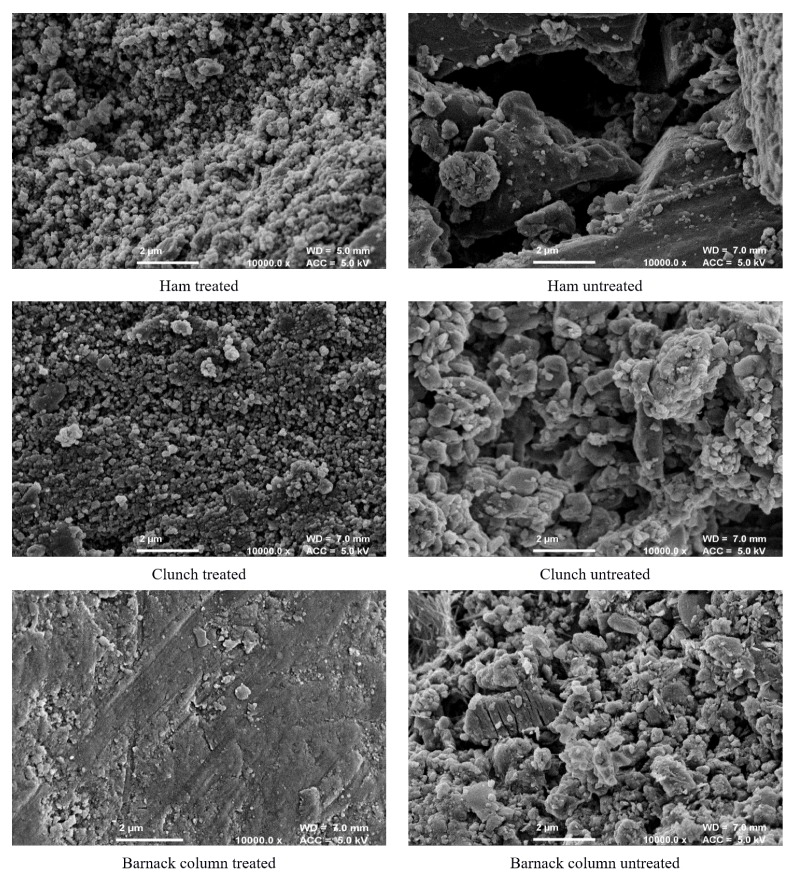
FESEM images of the Ham, Clunch and Barnack column stone specimens.

Figure 3 shows the FESEM images of untreated and treated surfaces of Ham, Clunch and Barnack column after 12 weeks from the treatment. The images highlight changes in the porosity of the stone specimens 4 years after the beginning of the test.

Images of the polished sections of the 6 specimens are reported in Figure 2a–f whereas results of the calculations carried out using binarised images of the same sections are reported in Table 2. Results suggest a reduction in porosity from the bulk to the surface in Portland and Clunch stone. Barnack ‘column’ and Ham are characterized by an increase in the porosity from the bulk to the surface, whereas Barnack ‘mould’ by a very low and consistent porosity through the whole section. Magnesian limestone is characterised by a decrease from the bulk to the middle section (section #2) and a subsequent increase from the middle section to the surface, with an overall small reduction between regions #3 and #1.

### 3.2. Drilling Resistance Measurements

**Figure 4:**
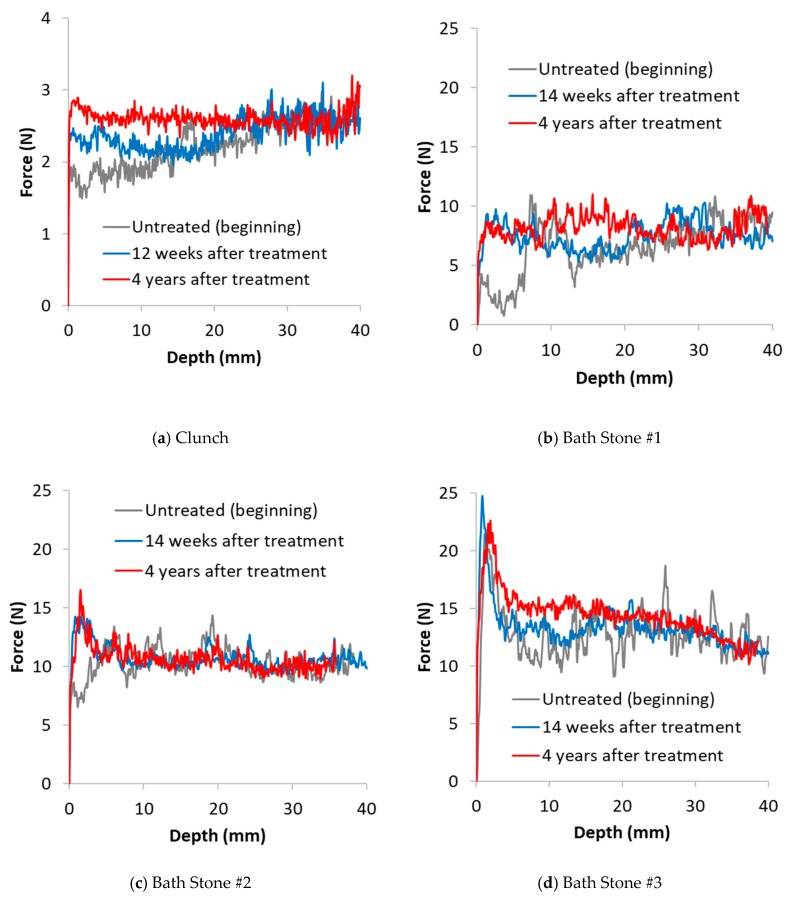
Drilling resistance vs. Drilling depth untreated (beginning), 12 weeks after treatment and 4 years after treatment of stone specimens where nanolime had a positive consolidating effect. (**a**) Clunch; (**b**) Bath Stone #1; (**c**) Bath Stone #2; (**d**) Bath Stone #3.

**Figure 5:**
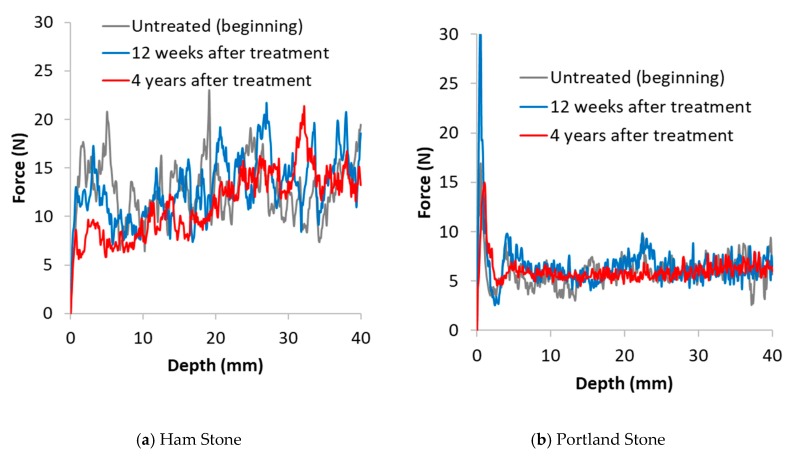
Drilling resistance vs. drilling depth for (**a**) the Ham stone and (**b**) Portland stone, untreated (beginning), 12 weeks and 4 years after treatment.

**Figure 6:**
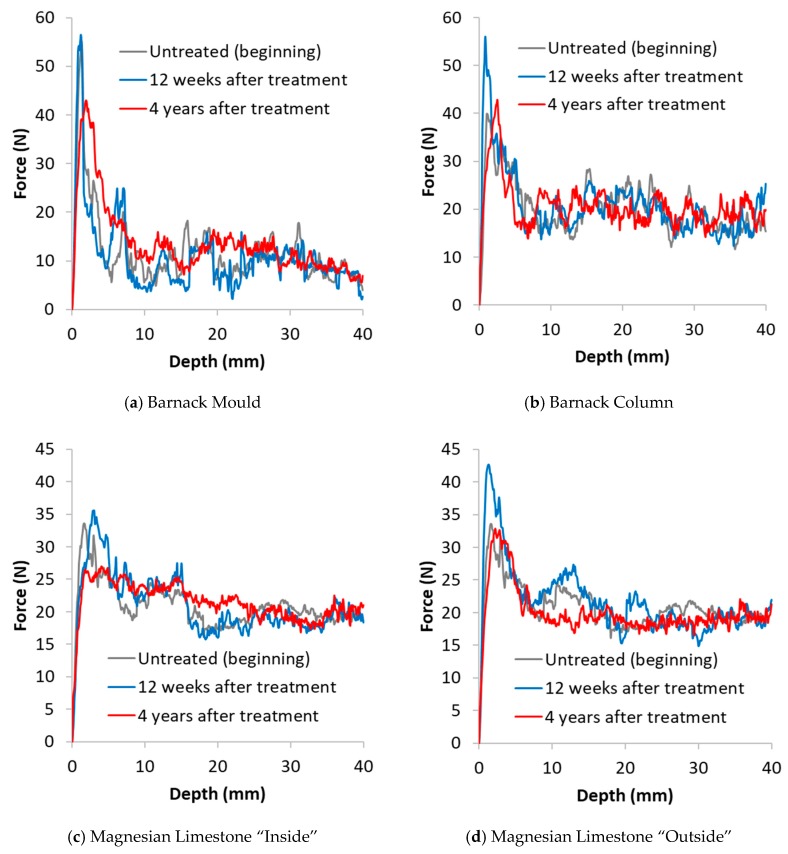
Drilling resistance vs. drilling depth untreated (beginning), 12 weeks after treatment and 4 years after treatment of stone specimens for which nanolime had a neutral consolidating effect. (**a**) Barnack Mould; (**b**) Barnack Column; (**c**) Magnesian Limestone “Inside”; (**d**) Magnesian Limestone “Outside”.

Results of the drilling resistance measurements are reported in Figure 4, Figure 5 and Figure 6. In these images, the DR curves of the treated and untreated specimens were aligned to correspond with the resistance at a depth unaffected by the treatment, typically 30 to 40 mm [2]. Each line is an average of the best 3 tests carried out on each surface. This allowed a meaningful comparison between treated and non-treated samples to be made. For a clearer comparison of the results, the tests are divided into 3 groups: (i) stones exhibiting behaviour consistent with the expected results of a consolidation treatment (i.e., positive effect) such as strengthening (Clunch and Bath stones; Figure 4a,b); (ii) stones where the application of nanolime had a negative effect (i.e., Ham stone; Figure 5a); (iii) stones where the application of nanolime had neither a positive nor a negative effect (i.e., Barnack, Portland and magnesian limestone; Figure 5b and Figure 6a–d).

Results of the DRMS on the untreated stones allowed identification of a crust of altered material on the surface of four lithotypes namely Bath (Figure 4c,d), Portland, Magnesian limestone (both samples) and Barnack stone (both samples; Figure 6a–d). Results related to Bath and Portland stones are supported by the observations of the polished sections in Figure 2. Results of the investigation on the porosity change suggest that for Portland stone this crust is connected to a reduction in porosity and, in particular, to the precipitation of exogenous material within the pores of the surface.

**Figure 7:**
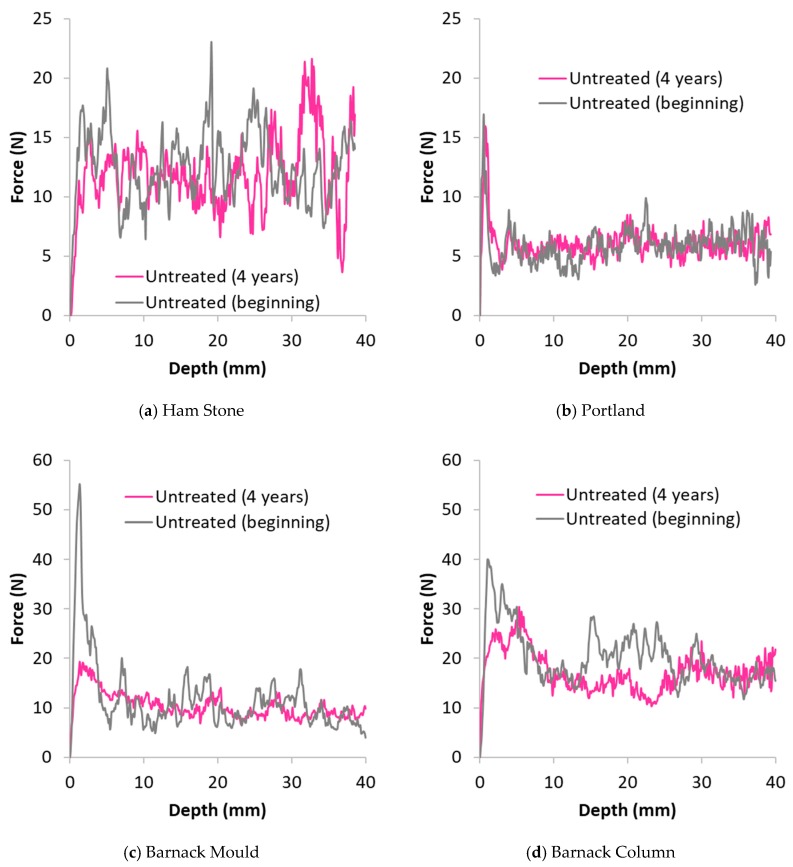
Drilling resistance vs. drilling depth of untreated stone specimens tested at the beginning and at the end of the test. (**a**) Ham Stone; (**b**) Portland; (**c**) Barnack Mould; (**d**) Barnack Column.

Figure 7 shows the DR of the untreated areas of the same stone at the beginning and at the end of the testing period for Ham, Portland and Barnack (mould and column) stones. Drilling resistance of the untreated area of the same stones at the beginning and end of the test show that only Barnack stone (both samples, Figure 7c,d) was affected by the weathering during the 4 years of the test whereas Ham and Portland stone do not show any effect of the weathering.

DRMS results corresponding to 12 weeks and 4 years for Clunch stone (Figure 4a) highlight an increase in drilling resistance over the time, which is visible up to a depth of about 20 mm from the surface. An increase in drilling resistance is also visible in 2 of the 3 specimens of Bath stone, although in this case the effects of nanolime are not always very clear and also the penetration depth is variable with the sample reaching a maximum of approximately 16 mm in sample #3.

Results for both samples of Barnack stone (Figure 6a,b) suggest an initial increase in drilling resistance after 12 weeks, followed by a subsequent reduction that, in the case of the ‘column’ sample, reaches lower values compared to the drilling resistance at the beginning of the test.

Penetration depth of nanolime is difficult to estimate in the ‘mould’ sample whereas for the ‘column’, it is possible to estimate a penetration of approximately 15 mm after 4 years.

As mentioned above, both samples of Barnack stone were characterized by a harder crust on the surface and this may have affected the penetration of nanolime. A similar trend can be observed for both samples of Magnesium limestone that, as well as Barnack, developed a harder crust on the surface. In this case it is difficult to estimate the penetration depth.

Ham stone (Figure 5a) shows a continuous reduction in drilling resistance over the 4 years of the tests with effects visible up to a depth of 6mm.

Portland stone (Figure 6c,d) show a dramatic increase in drilling resistance of the crust after 12 weeks and a similar reduction after 4 years when the surface crust became weaker compared to at the beginning of the test. Penetration depth after 4 years can be estimated to be about 3 mm however, in this case it is clear that the nanolime failed to reinforce the weaker zone of the stone visible immediately behind the crust.

### 3.3. Water Absorption Test

**Figure 8:**
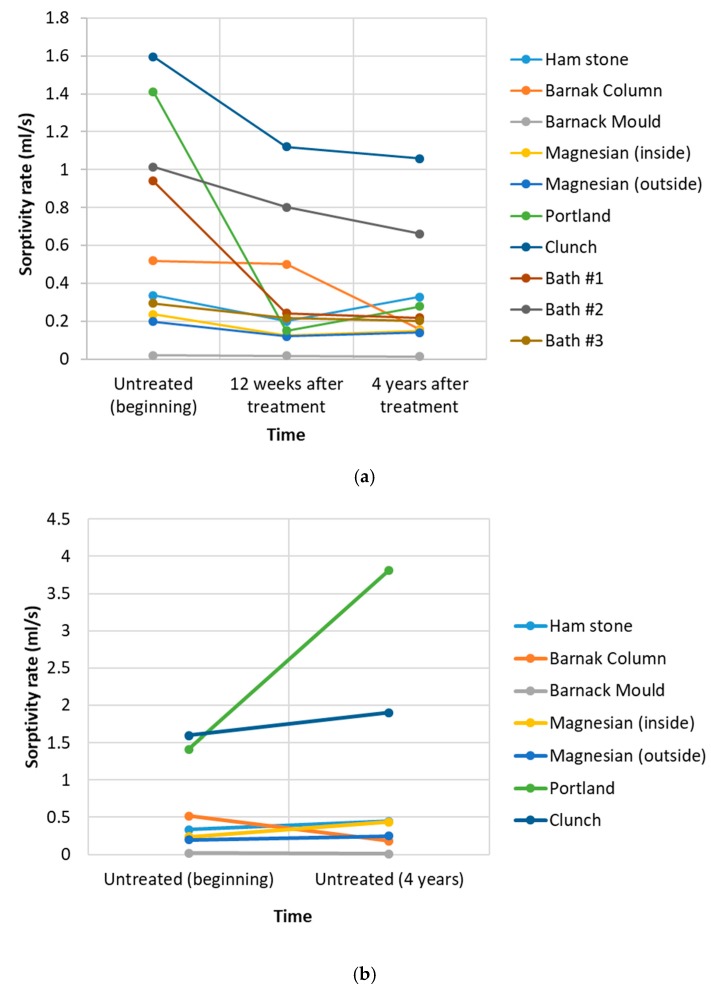
(**a**) Sorptivity rates of the various stone specimens untreated (beginning) and after 12 weeks and 4 years. (**b**) Comparison of the sorptivity rate of the untreated stone at the beginning and the end of the testing period (4 years).

Figure 8a shows the sorption rate of the untreated and treated samples at 12 weeks and 4 years following treatment. Results of the sorptivity on untreated areas at the beginning and at the end of the test (Figure 8b) suggest a small increase of sorptivity in Ham, Magnesian limestone (both samples) and Clunch, whereas Portland stone shows a dramatic increase. The only specimen showing a reduction in sorptivity was Barnack stone (both samples). Sorptivity of the Bath stone untreated material after 4 years is omitted because the specimens had an uneven surface that did not allow effective sealing of the Karsten tube.

## 4. Discussion

### 4.1. Effect of the Stone Type on the Effectiveness of the Treatment

Results of the drilling resistance and surface water absorption over the whole duration of these tests, suggest that the effects of nanolime as a stone consolidant depends on the stone type, with some stones (e.g., Clunch and Bath Stone) demonstrating positive changes, whereas other stones (e.g., Ham Stone) negative changes. Others stones again (e.g., Barnack, Magnesian limestone and Portland stones) showed an initial positive change that reduced over the 4 years of the study. A detailed discussion of the effects of nanolime for each group is reported in the following paragraphs.

#### 4.1.1. Negatively affected stones

Ham stone is the only stone that showed a decrease in drilling resistance of the treated area over the 4 years of the test. However, examination of Figure 7a reveals a decrease in the drilling resistance in the untreated areas of the stone that could be explained by a natural weathering. Therefore, caution must be exercised as the decrease observed in the nanolime-treated areas may have been a consequence of the natural weathering and occurred irrespective of the presence of the consolidant. Figure 8b shows the sorptivity of the treated and untreated parts of the stone 4 years after the treatment where an increase in the ease by which the water is absorbed is highlighted. Consideration of the stone porosity as shown in Table 2 indicates a very large difference between region #1 and region #2.

#### 4.1.2. Positively Affected Stones

In the case of Clunch and Bath stone, the application of nanolime not only increased the drilling resistance after 12 weeks from the treatment, but also had a long-lasting effect (Figure 4a,b). The result suggests that in both cases, the time for achieving a full consolidation effect was within the initial 12 weeks and that the increase in drilling resistance is not proportional to the time that passed by.

Overall, these results suggest that, over time, the nanolime has had a consolidation effect on the stones, up to the point where the original drilling resistance of the stone was fully restored. The penetration depth was found to be 12 mm after 12 weeks in the Clunch stone, and 20 mm after 4 years. It is possible that the absence of a surface crust in the Clunch stone offered greater opportunities for the nanolime crystals to penetrate inside the sample.

Results of the absorption test for the three Bath stone specimens show a significant decrease in the sorptivity rate 12 weeks after the treatment (Figure 7a), probably due to the effect of the carbonation products filling the pores of the stone. The results 4 years later are almost identical in specimen #1 and specimen #3, suggesting that even if further weathering of the stone took place, the consolidant was subject to the same degradation process of the original matrix. Conversely, in the case of specimen #2, the trend shows a continuous decrease.

#### 4.1.3. Neutrally Affected Stones

This group includes all stones where the consolidating effect of nanolime was no longer detectable 4 years following the treatment. These include Barnack stone, Portland stone and Magnesium limestone. As demonstrated by the SEM images and the DRMS results, in these samples a surface crust was detected before any treatment. It is likely that this naturally formed crust influenced the penetration of nanolime inside the samples and, therefore, the effectiveness of the treatment (although Bath stone was found with a similar crust but the effect of nanolime was positive overall). 

In all cases, the treatment has increased the drilling resistance of the crust over the short-term (i.e., 12 weeks), whereas after 4 years, the effect reduced to the point that the drilling resistance of the crusts returned to values similar to those measured before the treatment. A comparison of the SEM images of treated and untreated low density Barnack stones suggest that the treatment almost completely blocked the stone pores. A decrease in DR of stone surfaces treated with nanolime was observed also by other authors [31] who suggested that, substrates treated with nanolime may require a periodic application of this consolidant to achieve long term effects on their mechanical and physical properties.

### 4.2. Effect of the Environmental Conditions on the Effectiveness of the Treatment

As previously mentioned, the sample of Magnesium limestone used in this project, was divided into two pieces: initially a piece was stored in an outside sheltered location (Magnesian limestone #2), whereas the other piece in a conditioning room (Magnesian limestone #1). Interestingly, over the initial 12 weeks the stone located outside showed a greater increase in drilling resistance compared to the stone inside. This result is supported by the results published by Lopez-Arce and colleagues [18] suggesting that precipitation and transformation of CaCO_3_ polymorphs are highly dependent on the relative humidity and exposure time (e.g., higher RH promotes formation of calcite and aragonite). In both cases (i.e., sample stored indoor and outdoor) the drilling resistance of the crust seems to reduce with the time of the test. Overall, our results are supported by the literature and confirm the substantial effect of the environmental condition on the nanolime’s consolidation effects. 

### 4.3. Effect of the Pre-Treatment on the Effectiveness of the Consolidation Process

As highlighted in Table 1, Bath stone was treated following 3 different protocols: specimen #1 was pre-treated with distilled water prior to nanolime application; specimen #2 was not pre-treated, whereas specimen #3 was pre-treated with ethanol. As illustrated in Figure 4b–d, in the sample pre-wet with water (sample #1, Figure 4b) the drilling resistance increased up to a depth of 7 mm from the sample surface, whereas the sample that wasn’t pre-wetted (Figure 4c) showed an increase in drilling resistance up to a maximum of 4 mm. The original drilling resistance of both samples was restored within the initial 12 weeks since application, and this value was maintained for the following 4 years. The sample pre-wetted with ethanol (sample #3, Figure 4d), showed the existence of a surface crust that can be the cause of a very minimal penetration depth to which the increase of drilling resistance can be observed. Nevertheless, 4 years after treatment, the drilling resistance was observable up to an impressive depth of 14 mm. This result does not seem to support the ethanol pre-wetting theory, which states that as time passes, ethanol would evaporate relatively fast drawing nanolime suspended particles back to the surface [19].

## 5. Conclusions

An investigation was carried out to determine how stone type influences the effectiveness of nanolime as a consolidation treatment after 12 weeks and 4 years. In addition, the effect of environmental conditions and pre-treatments on the characteristics of the consolidation are also reported.

Our results demonstrate for the first time that, over a period of time of 4 years, the nanolime treatment can have either a positive, a negative or variable effects on the mechanical characteristics of stones. This can be attributed to a strong correlation between the effectiveness of the treatment and the physical and chemical characteristics of the stones such as porosity and mineralogical composition. The porosity, for instance, may have a substantial influence on the amount of nanolime actually absorbed by the stone, which is different compared to the amount of nanolime applied, since part of the nanolime used during the treatment runs off the surface and accumulates at the bottom. Differences between the results obtained with various stones may be related to this factor, however, this was not the focus of this research and it is very difficult to evaluate on-site.

A comparison of the absolute values of the drilling resistance for the weaker, finely porous stone samples, such as Clunch and Bath stone suggests that these lithotypes benefited most from the application of the nanolime over 12 weeks and 4 years. In such cases, our results suggest that the use of nanolime as a consolidant can stabilize the stone and help in preventing further deterioration.

In contrast, for lithotypes such as Barnack, Portland and Magnesium limestone, the nanolime treatment has not showed a clear beneficial effect. Borsoi and colleagues [19] stated that in coarse porous calcareous stones, nanolime tends to accumulate beneath the surface, impairing the quality of the consolidation treatment and obstructing further penetration.

This research is of immense importance and value to conservators as it shows the use of nanolime as a consolidant for weathered stones can be beneficial for certain types of stones but not for all lithotypes. Our research provides guidance on how the effectiveness of nanolime treatments can be influenced by the application method of this consolidant (e.g., pre-wetting with water). This will bring great impact to the conservation industry by providing the much needed data to allow conservators to customise the treatment methodology for a specific building (stone type). This will directly benefit the conservation of heritage buildings constructed from limestones, including and equivalent to, those of the study.

## Figures and Tables

**Table 1:** Nanolime treatments applied to the stone specimens.

Stone Specimen	Pre-Wetting	Treatment Details
Clunch	No	6 applications of E-25 in 6 days. Stored in a conditioned chamber at 25 °C and 65% RH
Bath Stone #1	Water	
Bath Stone #2	No	
Bath Stone #3	Ethanol	
Barnack ‘column’	No	
Barnack ‘mould’	No	
Portland Stone	No	
Ham Stone	No	
Magnesian limestone #1	No	
Magnesian limestone #2	No	6 applications of E-25 in 6 days. Stored in a sheltered outdoor environment

**Table 2:** Stone porosities (%) calculated after 12 weeks from the treatment, using black-to-white binarisation of polished stone cross-sectional images using imageJ.

Porosity (%)	Barnack ‘Column’	Barnack ‘Mould’	Clunch	Ham	Magnesium	Portland
Region #1 (surface)	19.9	0.3	19.8	6.5	14.0	29.2
Region #2	13.9	0.3	24.2	0.7	8.7	30.6
Region #3 (bulk)	9.2	0.3	-	-	15.3	32.1
